# LKC-Net: large kernel convolution object detection network

**DOI:** 10.1038/s41598-023-36724-x

**Published:** 2023-06-12

**Authors:** Weina Wang, Shuangyong Li, Jiapeng Shao, Huxidan Jumahong

**Affiliations:** 1 College of Information and Control Engineering, Jilin Institute of Chemical Technology, Jilin, 132000 China; 2 School of Network Security and Information Technology, YiLi Normal University, Yining, 835000 China

**Keywords:** Engineering, Mathematics and computing

## Abstract

Deep learning-based object detection methods have achieved great performance improvement. However, since small kernel convolution has been widely used, the semantic feature is difficult to obtain due to the small receptive fields, and the key information cannot be highlighted, resulting in a series of problems such as wrong detection, missing detection, and repeated detection. To overcome these problems, we propose a large kernel convolution object detection network based on feature capture enhancement and vast receptive field attention, called LKC-Net. Firstly, a feature capture enhancement block based on large kernel convolution is proposed to improve the semantic feature capturing ability, and depth convolution is used to reduce the number of parameters. Then, the vast receptive filed attention mechanism is constructed to enhance channel direction information extraction ability, and it is more compatible with the proposed backbone than other existing attention mechanisms. Finally, the loss function is improved by introducing the SIoU, which can overcome the angle mismatch problem between the ground truth and prediction box. Experiments are conducted on Pascal VOC and MS COCO datasets for demonstrating the performance of LKC-Net.

## Introduction

Object detection is an important task in the field of computer vision, and it is also essential to be employed in other advanced visual tasks, such as behavior recognition^1^, attitude estimation^2^, and video segmentation^3^. The task of object detection is to use RGB images as input and realize location annotation, classification of interest targets, and display the confidence of their categories. Object detection has been widely used in many fields, including traffic detection^4,5^, medical detection^6,7^, industrial detection^8,9^, and many others.

The receptive field is an important design element for object detection. To expand the receptive field, researchers usually used a relatively large convolution kernel in the network model, so that the model could obtain more comprehensive features of the input image, such as LeNet(5*5)^10^ and AlexNet(5*5, 11*11)^11^. A large number of object detection networks based on large kernel convolution, including effective receptive field (ERF)^12^, RepLKNet^13^, and ViT^14^, have been proposed. Nevertheless, the incorporation of large kernel convolutions can bring some associated challenges. First, the inappropriate position of large kernel convolution may degrade the performance of the network. Second, it is difficult to determine the size of large kernel convolution that can achieve the best performance. Third, the introduction of large kernel convolution will result in a significant increase in the number of parameters and computation costs in the network. Therefore, this paper aims to determine the optimal position and size of large kernel convolution and reduce the number of parameters while maintaining the prediction performance.

**Figure 1:**
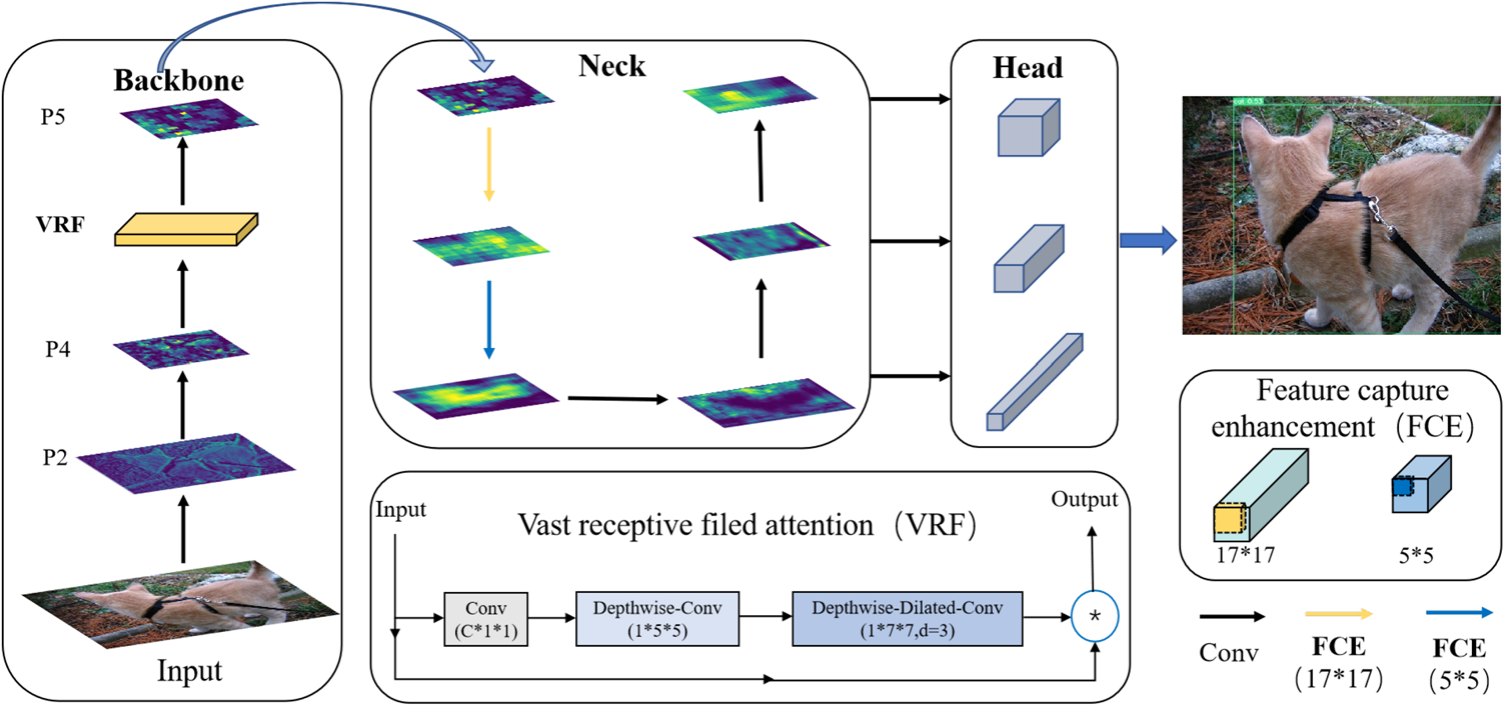
The overview of LKC-Net.

To address the above consideration, the ultimate goal of this paper is to propose an object detection network with a large kernel convolution block (LKC-Net). Firstly, a feature capture enhancement block based on large kernel convolution is proposed. The 1*1 convolution blocks in the neck’s bottom-up fusion feature convolution modules are replaced with 17*17 and 5*5 convolution blocks, respectively. The standard convolution is replaced with depthwise convolution to reduce network parameters and improve network efficiency. Then, the attention mechanism with a large receptive field is fused in the backbone to make the whole model more suitable for the large convolution kernel structure. Finally, the loss function is improved by introducing SIoU. The distance loss is modified, and angle loss is added to further improve the performance of network detection. The overview of the proposed network LKC-Net is shown in Fig. 1.

The contributions can be summarized as follows: (a) The feature capture enhancement block is designed to improve the feature capturing ability of the neck. The kernel size of the convolution blocks in the neck is enlarged to obtain a larger receptive field, and the depth convolution is used to reduce the number of parameters. Compared with the existing models, the proposed network has higher accuracy with the same number of parameters. (b) The vast receptive field attention is constructed to enhance the channel direction information extraction ability. The model integrates the attention mechanism with a large receptive field more compatible with the proposed backbone. Therefore, the detection accuracy can be further improved under the combined action of the large convolution neck and the large receptive field attention mechanism. (c) The loss function is improved by introducing the SIoU, which can overcome the angle mismatch problem between the ground truth and prediction box. This can effectively enhance the computational performance of the detection model. (d) The object detection model with a large kernel convolution block is proposed. The large kernel convolution neck structure integrates high-level and low-level features, which can improve the ability to extract semantic information features of image context. Therefore, the performance of network detection is enhanced. (e) Extensive experiments have been carried out on the Pascal VOC and MS COCO datasets, proving the advantages of LKC-Net over the existing methods by quantitative and qualitative evaluation.

The main structure of this paper is as follows: “Related works” introduces the related works to large kernel convolution and attention mechanism. In “Proposed method”, the objection detection model is proposed, and the main components are described in detail. A series of experiments and visualization is performed in “Experiment”. Conclusions are presented in “Conclusion”.

## Related works

### Overview of object detection

In recent years, object detection models have been mainly divided into two categories: two-stage detection model and one-stage detection model. The process of the two-stage object detection model is divided into two steps. In the first step, the candidate region is extracted from the input image. In the second step, the candidate region is send to the CNN network for detection. Classical two-stage object detection models include R-CNN^15^, Faster R-CNN^16^, etc. The two-stage model has been the leader in the field of object detection for a long period, but the fatal disadvantage of this kind model is that the detection speed is not efficient enough. Although some two-stage models have improved the detection speed, they still cannot meet the requirements of real-time detection. The one-stage model breaks the dominance of the two-stage model in terms of detection speed. With the advent of YOLO^17^ in 2015, the one-stage object detection model began to boom. Its basic idea is to divide the input image into $S*S$ grids, and each grid predicts *B* bounding boxes. Then, the input image is send into the neural network to extract features. Finally, the network directly predicts and outputs the detection result. There are other one-stage object detection networks such as SSD^18^ and RetinaNet^19^. However, these models did not evolve as well as YOLO in subsequent versions, and YOLOV5 model is chosen as our baseline.

### Large kernel convolution

The receptive field is an important element for object detection neural network models. The receptive field of a convolutional neural network unit corresponds to the fixed region in the image of the previous layer, and the image outside the corresponding region of the receptive field cannot affect the unit. The larger the receptive field the neural network unit owned, the more context information of the image received by the convolutional neural network unit. Therefore, enabling the network to extract features from images on a larger vision can be more sensitive to input images. There are many methods that use large kernel convolution to improve the receptive field of networks, such as LeNet(5*5)^10^, AlexNet(11*11)^11^, Inception(1*7, 7*1)^20^, GoogLeNet(7*7, 5*5)^21^. Chen. et al^22^also find that the large kernel convolution has excellent performance not only in 2D CNNs model but also in 3D CNNs.

Although large kernel convolution can better obtain contextual information on detection targets, some researchers have found that the size of the convolution kernel is not that the larger convolution kernel size leads to better model performance. Sheng et al.^23^ proposed that only local features can be observed if the receptive field is too small, and the relationship between features can be ignored. If the receptive field is too large, too much invalid information will be retrieved, decreasing the representation ability of the network. Han et al.^24^ tried the convolution kernel size of 7*7 and 9*9 in the segmentation task, and found that the 7*7 convolution kernel would improve the network. However, the 9*9 convolution kernel would cause performance degradation. Therefore, large kernel convolution of different sizes may affect downstream tasks differently.

This paper verifies the influence of convolution kernels with different sizes. On this basis, a context enhancement block is proposed, which can effectively enlarge the receptive field of the detection model, and it is combined with the YOLOV5 object detection model to improve the detection precision.

### Attention mechanism

The attention mechanism originates from the study of the human visual system. When humans observe things with their eyes, they do not focus on everything in their visual field but selectively look at the part of their visual field that they want to get information from. Inspired by this observation, researchers have designed different attention mechanisms for various tasks to enhance the network’s attention to the target of interest. At present, the existing attention mechanisms include Squeeze-and-Excitation (SE)^25^, Convolutional Block Attention Module (CBAM)^26^, and Channel Attention (CA)^26^, etc. There are also some attention mechanism used for distill detection model to fusion different modality, such as modality attention-based fusion (MAF)^27^.

However, the experimental verification shows that the use of some attention mechanisms can not improve the network’s detection effect but reduces the network’s accuracy. This indicates that some existing attention mechanisms are not in harmony with large kernel convolution and even hinder the improvement of detection performance.

In this paper, the attention mechanism with a large receptive field is introduced to fit the detection network model of large kernel convolution. Under the combined action of the large convolution neck and the large receptive field attention mechanism, the network model can enhance the ability to extract context information from an image, thereby improving the detection accuracy of the network.

### Loss function

The loss function of the YOLO series is composed of three kinds of losses, namely, box location loss, classification loss, and confidence loss. For box location loss, YOLO usually adopts IoU series losses, which have experienced from IoU loss to Generalized-IoU loss (GIoU)^26^, Distence-IoU loss (DIoU)^28^, Conplete-IoU loss (CIoU)^28^. In version 6.0 of YOLOV5, CIoU is used for IoU loss of positioning frame. Compared with DIoU, CIoU added the influence factor of the size of the detection frame and further considered the aspect ratio between the bounding box and the ground truth, making the prediction frame closer to the ground truth.

However, CIoU loss does not consider the angle problem between ground truth and the bounding box. The angle mismatch may cause the restriction for the prediction box in the training process and eventually lead to the training model with poor performance. Therefore, the SIoU is introduced into the proposed network to increase the angle matching between the bounding box and ground truth for improvement performance.

## Proposed method

**Figure 2:**
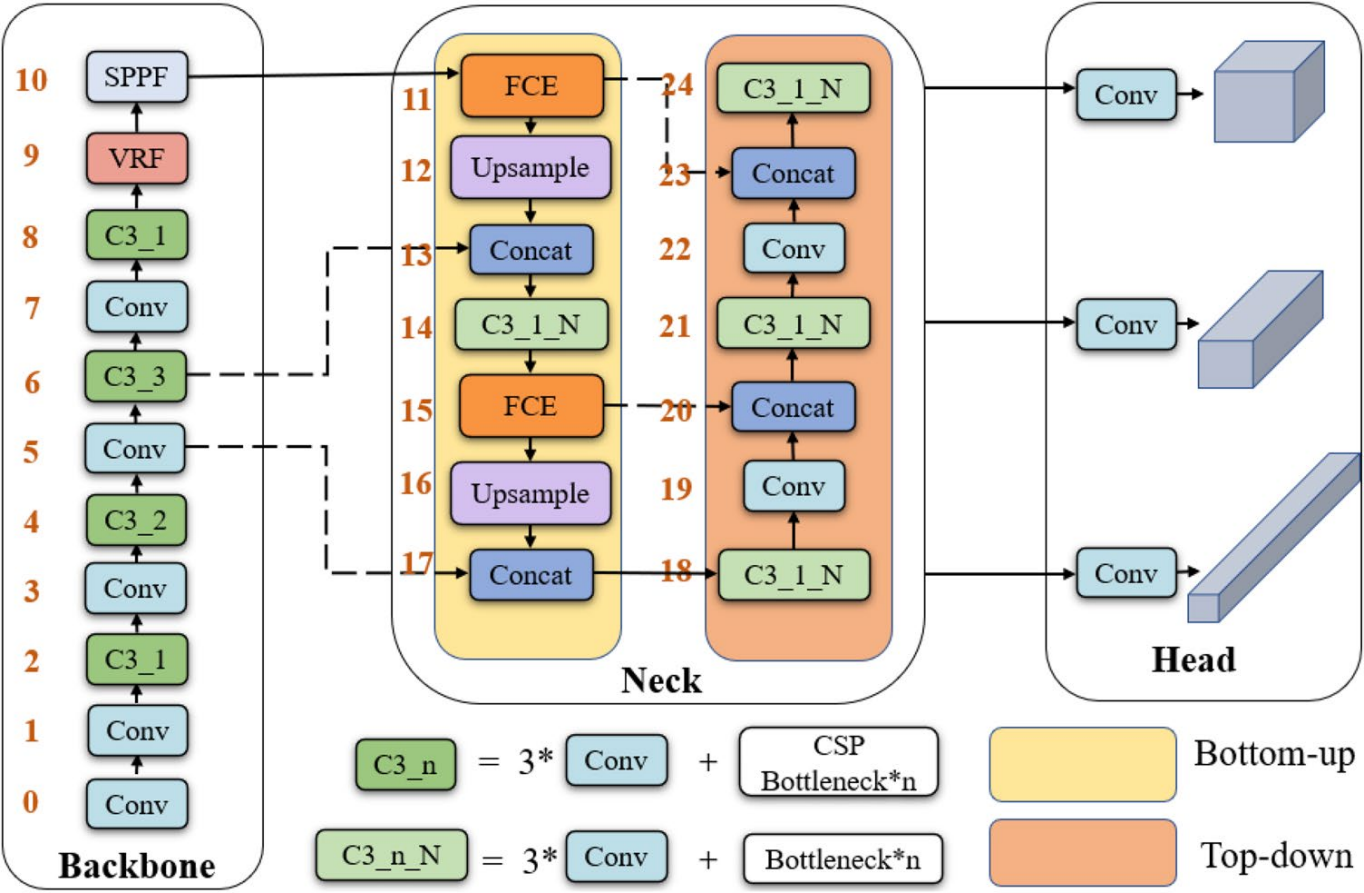
The Structure of LKC-Net.

In this section, the object detection model with large kernel convolution based on feature capture enhancement and vast receptive field attention (LKC-Net) is proposed. Firstly, the feature context enhancement (FCE) block based on large kernel convolution is proposed. It is integrated into the neck network to enlarge the receptive field of the neck and enhance the neck’s ability to extract high-level feature context information in the bottom-up process. Then, the vast receptive field (VRF) attention mechanism is constructed to strengthen the network’s attention to extracting features in a larger receptive field. Finally, the loss function is improved by introducing the SIoU, which can overcome the angle mismatch problem between the ground truth and prediction box. The general structure of LKC-Net is illustrated in Fig. 2. The main components of the proposed model will be presented in what follows.

### Baseline

The baseline adopts the basic architecture of the YOLOV5s^29^ model. YOLOV5 is an end-to-end object detection model with different parameter sizes: S, M, and L, for different purposes. The network has been updated from version 1.0 to version 6.0. The whole network consists of three parts: the backbone, neck, and head. The backbone is used to extract the features of input images. The neck is used to integrate the high-level and low-level features extracted from the backbone network and thus strengthen the ability of the whole network to extract image features. The head is used to predict and output the result of the detection. The loss function used by YOLOV5 in version 6.0 is CIoU. Compared with DIoU^28^, CIoU considers the consistency of the aspect ratio of the three regression elements in the bounding box, which further improves the detection accuracy of the network. The CIoU loss function is defined as follows:

1 $$\begin{aligned} L_{CIoU}= & {} 1-\textrm{IoU}+\frac{\rho ^2(b,b^{gt})}{c^2}+\upsilon \end{aligned}$$

2 $$\begin{aligned} \alpha= & {} \frac{\nu }{1-\textrm{IoU}}+\upsilon \end{aligned}$$

3 $$\begin{aligned} \upsilon= & {} \frac{4}{\pi ^2}(\arctan \frac{w^{gt}}{h^{gt}}-\arctan \frac{w}{h})^2 \end{aligned}$$

where IOU is the intersection ratio between the ground truth and the bounding box, $\rho (\cdot )$ is the Euclidean distance, and *c* is the diagonal line containing the smallest box. $\alpha$ is the weight function and is used to measure the consistency of the aspect ratio. It can be seen from the definition of $\alpha$ that the CIoU loss tends to be optimized in the direction of increasing the overlap area.

### Feature capture enhancement block

The high-level and low-level features extracted from the backbone network have different characteristics. The low-level feature contains less semantic information, but the information provided by the low-level feature is significant for predicting the object’s location. Compared with the low-level features, the high-level features contain richer semantic information. The prediction based on high-level semantic information can better identify the object’s content, but it is difficult to accurately predict the object’s location. In the neck network of the original YOLOV5 model, the strong semantic features extracted from the backbone are conveyed to the bottom-up structure, and the high-level semantics are integrated with the low-level semantics. Then, convolution is used to extract the fused features in the top-down path. Finally, the head outputs the result of the detection. In the neck of the original YOLOV5, the size of the bottom-up structure convolution kernel is 1*1. This part is mainly used to fuse the feature map extracted from the backbone network, and the 1*1 convolution is utilized to extract the spatial features in high latitudes.

**Figure 3:**
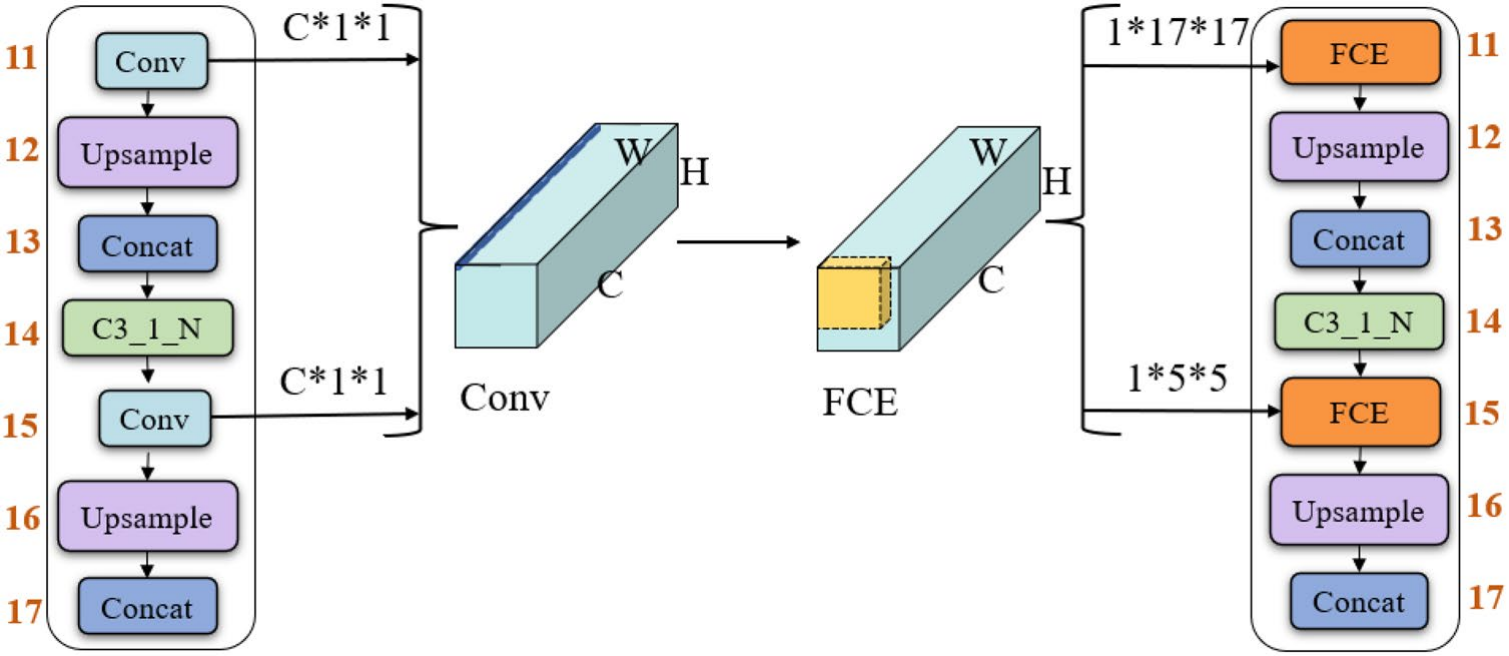
The feature capture enhancement block.

However, the small kernel convolution results in the loss of contextual semantic information. To overcome this shortage, the convolution kernel size of bottom-up feature extraction is enlarged to improve the network’s ability to extract high-level features and strengthen the network’s common recognition of background and object information. Based on the above-mentioned consideration, the feature capture enhancement block (FCE) is proposed. FCE enlarges the size of the convolution kernel for extracting high-level semantic information in the neck network, i.e. the 1*1 convolutions are replaced by the 17*17 and 5*5 convolutions, respectively. FCE can enhance the feature capture ability of the original PANet bottom-up module. Furthermore, the original convolution mode is modified to the depth convolution. This not only increases the receptive field of the network but also reduces the parameters of the network. The feature capture enhancement block is shown in Fig. 3.

The number of parameters is reduced by utilized of the depth convolution. The analysis of the number of parameters in depthwise convolution and standard convolution is performed as follows. Let $D_K$ be the size of the convolution kernel, *M* be the number of the feature map’s channels at the input end, *N* be the number of feature map channels at the output end, and $D_F$ be the size of the feature map at the output end. The comparison of the parameter number of the two parts is as follows:

4 $$\begin{aligned} \frac{D_K\cdot D_K\cdot M\cdot D_F\cdot D_F}{D_K\cdot D_K\cdot M\cdot N\cdot D_F\cdot D_F}=\frac{1}{N} \end{aligned}$$

It can be seen that the number of parameters in FCE block decreases *N* times, where *N* is the number of channels in standard convolution. Thus, the FCE block effectively reduces the number of parameters and improves network efficiency.

### Vast receptive field attention

**Figure 4:**
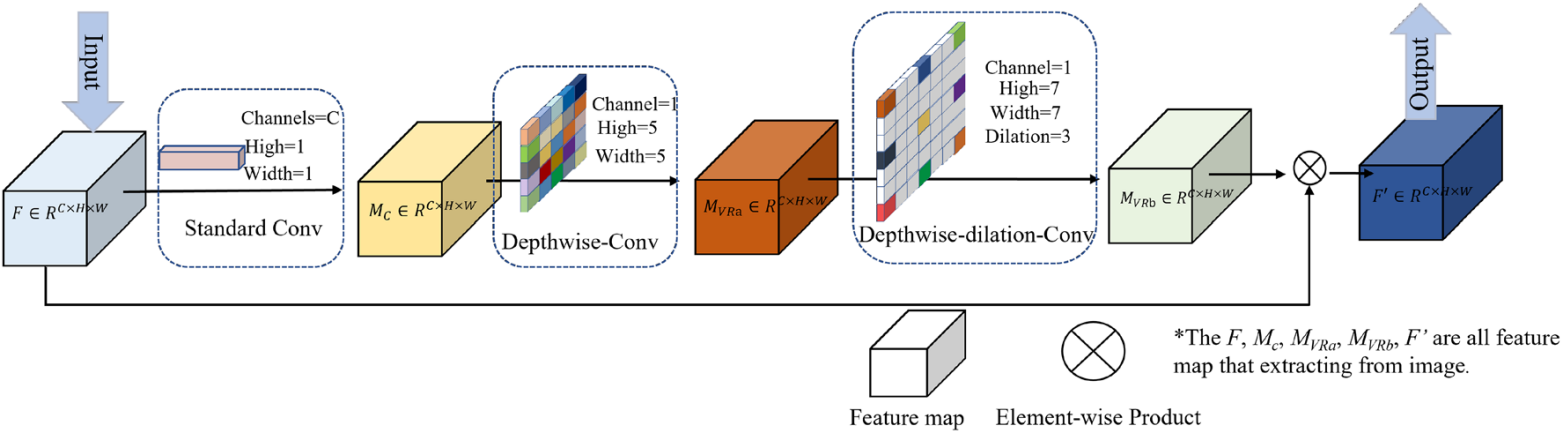
The vast receptive field attention.

In the process described above, the feature capture enhancement block can increase the receptive field and reduce the number of network parameters. This improvement may lead to the loss of the channel information, thus attention mechanism is added to obtain channel direction information. At present, some well-known attention mechanisms can enhance the ability of the network to obtain spatial information, such as SE^25^, CBAM^26^, and CA^26^. However, the experimental verification shows that the use of these attention mechanisms not only cannot improve the detection effect of the network but also reduces the accuracy of the network. The above attention mechanisms make the network backbone search for the target of interest in the limited receptive field, which leads to conflict with the neck network with large convolution features, resulting in an unsatisfactory result. Therefore, the vast receptive field (VRF) attention is constructed to increase the receptive field and promote the performance of the neck network with large convolution features. The structure of vast receptive field attention is shown in Fig. 4.

Assuming the intermediate feature map $F\in R^{C\times H\times W}$ as the input, the VRF attention mechanism first uses a convolution block to calculate the feature map $M_C\in R^{C\times H\times W}$ that containing channel information. Then, the feature map is convolved twice to successively calculate the sum of two feature maps $M_{VRa}\in R^{C\times H\times W}$ and $M_{VRb}\in R^{C\times H\times W}$ with large receptive fields. Finally, the calculated attention feature map $M_{VRb}\in R^{C\times H\times W}$ is multiplied by the input intermediate feature map to obtain the enhanced feature map $F'\in R^{C\times H\times W}$. The overall process can be summarized as follows:

5 $$\begin{aligned} F'=F\otimes M_{VRb} ( M_{VRa} ( M_C (F))) \end{aligned}$$

where $F'$ is the final output feature map, and $\otimes$ is the element-wize multiplication. $M_C (F)$, $M_{VRa}(F)$, and $M_{VRb}(F)$ are the three convolution modes in VRF, which are defined as follows:

6 $$\begin{aligned} M_C (F)= & {} f^{C\times 1\times 1}(F) \end{aligned}$$

7 $$\begin{aligned} M_{VRa} (F)= & {} f^{ 5\times 5}_{DW}(F) \end{aligned}$$

8 $$\begin{aligned} M_{VRb} (F)= & {} f^{7\times 7}_{DWd}(F) \end{aligned}$$

where $f^{C\times 1\times 1}(F)$ represents the convolution whose convolution kernel is 1*1*C, $f^{ 5\times 5}_{DW}(F)$ represents the convolution calculation whose convolution kernel is 1*5*5, and $f^{7\times 7}_{DWd}(F)$ represents the deep cavity convolution whose convolution kernel is 1*7*7.

### Loss function

The loss function in YOLOV5 comprises three parts: classification loss, location loss, and confidence loss. Among them, the classification loss refers to whether the prediction box and the corresponding classification are correct, the positioning loss refers to the error between the bounding box and GT, and the confidence loss refers to the confidence of the target detected by the network. The loss function is defined as follows:

9 $$\begin{aligned} L=\sum _{k=0}^K{I^{obj}_{kij}} [\beta ^{balance}_k\alpha _{box}\sum _{i=0}^{S^2}\sum _{j=0}^Bl_{IoU}+ \alpha _{obj}\sum _{i=0}^{S^2}\sum _{j=0}^Bl_{obj}+ \alpha _{cls}\sum _{i=0}^{S^2}\sum _{j=0}^Bl_{cls}] \end{aligned}$$

where *K*, $S^2$ and *B* are the index of the output feature map, the index of the cell on the feature map, and the index of the anchor on each cell, respectively. $\alpha$ is the hyperparameter loss weight set for each loss. $I^{obj}_{kij}$ represents the $i_{th}$ cells and $j_{th}$ anchor in feature map, whose value is 1 if it is positive, and 0 if not. $\beta ^{balance}_{K}$ is the weight for balance three scales (80*80, 40*40, 20*20) feature map.

**Figure 5:**
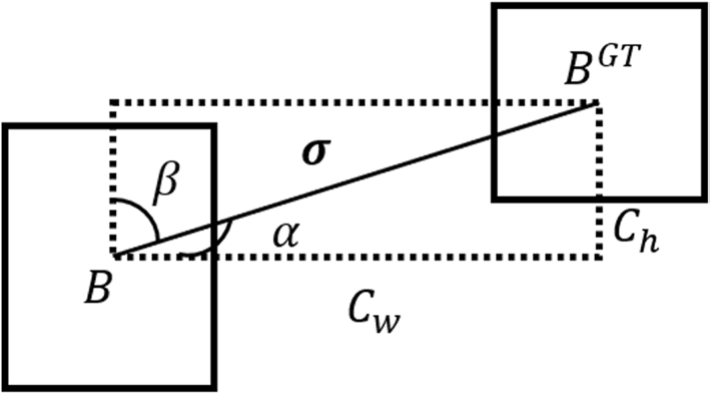
The angle loss of SIoU.

To overcome the angle mismatch problem between ground truth and bounding box, the loss function is improved by introducing the SIoU. The loss function is composed of four parts: angle, distance, shape, and IoU costs. The total box loss is as follows:

10 $$\begin{aligned} L=1-\textrm{IoU}+\frac{\Delta +\Omega }{2} \end{aligned}$$

where $\Delta$ is distance loss, $\Omega$ is shape loss, and IoU is IoU loss. The loss function redefines distance loss and considers angle loss $\Lambda$ into distance loss as follows:

11 $$\begin{aligned} \Delta = \sum \nolimits _{t=x,y}({1-e^{-\gamma \rho _t}}) \end{aligned}$$

12 $$\begin{aligned} \rho _x= & {} \left( \frac{b^{gt}_{cx}-b_{c_x}}{c_w}\right) ^2, \rho _y=\left( \frac{b^{gt}_{cy}-b_{c_y}}{c_h}\right) ^2, \gamma =2-\Lambda \nonumber \\ \Lambda= & {} 1-2*\sin ^2\left( \arctan (x)-\frac{\pi }{4}\right) , x=\frac{c_h}{\sigma }(\alpha ) \end{aligned}$$

where *x* is the hypotenuse for the connection $\sigma$ which between the center points of the anchor box *B* and the ground true $B^{GT}$, and the vertical distance $C_h$ is the sine value of the opposite side, as shown in Fig. 5.

## Experiment

In this section, the experiments are conducted to prove the effectiveness of the proposed model through quantitative and qualitative evaluation. The experiment is divided into four parts: (1) the experimental dataset and training environment configuration are introduced. (2) The quantitative evaluation is performed to verify the improvement of LKC-Net in accuracy on the Pascal VOC and MS COCO datasets. (3) The validity analysis and ablation experiment is performed to verify the effect of three innovative points in LKC-Net. (4) The qualitative evaluation is carried out to verify the improvement of LKC-Net in vision.

### Introduction of datasets

#### Pascal VOC dataset

**Figure 6:**
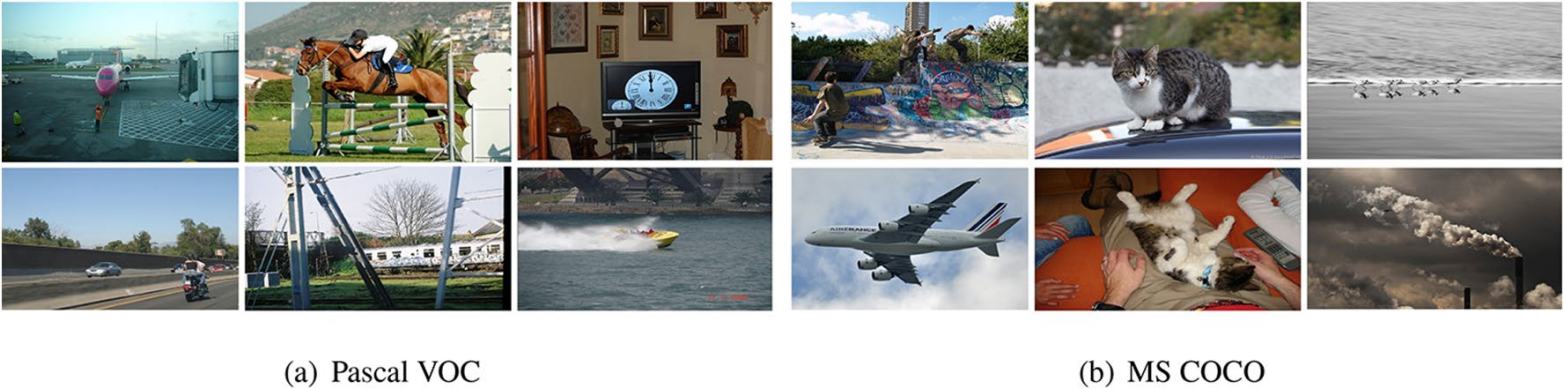
The representative pictures of Pascal VOC and MS COCO.

The PASCAL VOC Challenge^30^ consists of the following categories: Image Classification, Object Detection, Object Segmentation, Action Classification, etc. There are 20 main target categories in the Pascal VOC dataset. This paper mainly uses the object detection task data set of the VOC2007+2012 dataset for training, in which the train set contains 16,551 pictures, and the test set contains 4952 pictures. The representative pictures of the data set are shown in Fig. 6a.

#### MS COCO dataset

MS COCO^31^ is a very high industry status and large-scale dataset used for object detection, segmentation, image description, and other scenes. The dataset used in this paper is COCO2017, in which 80 categories of images are used for object detection. Dataset images are divided into train, verification, and test sets. There are 118,287 pictures in the train set, 5000 pictures in the verification set, and 40,670 pictures in the test set. The representative pictures of the data set are shown in Fig. 6b.

#### Experimental environment and hyperparameter settings

The experimental environment is PyTorch deep learning library, in which Pytorch version is 1.12.1+cu113, the version of torchaudio is 0.12.1+cu113, the version of torchextractor is 0.3.0. The version of torchvision is 0.13.1+cu113. The experiment is conducted on 12th Gen Intel(R) Core(TM) i7-12700@ 2.10GHz CPU, 32GB RAM, and NVIDIA RTX 3090 Ti GPU. The system is Windows 10 Pro version 19044.1826. The experimental hyperparameter settings are shown in Table 1.

**Table 1:** Hyperparameter settings.

Hyperparameter name	Number
Number of epoch	300
Batch_size	16
Input size	640
Optimizer	SGD
Initial learning rate	1e−2
Momentum	0.937
Weight_decay	5e−3
Weight_decay	5e−3
Warmup_epoch	3

### Quantitative evaluation

#### Experiment on Pascal VOC datasset

In the training process of the Pascal VOC dataset, the weight of pre-training on the COCO dataset is chosen. The train and verification set of VOC2007 and VOC2012 is used for the model training. The final results is tested on the VOC 2007 test set. The proposed model LKC-Net is compared with Fast YOLO^18^, Faster R-CNN VGG-16^18^, ShuffleNetV2-SSDLite^32^, RefineDet512-VGG-16^33^, RFB Net512-VGG^33^, MobileNetV2-YOLOV4^34^, EEEA-Net-c2- yolov4^34^, SSD300^18^, YOLOV5s^29^, YOLOV6-N^35^, and YOLOV7-Tiny^36^. The detection result on the VOC dataset is shown in Table 2.

**Table 2:** Comparison with different models on Pascal VOC dataset.

Models	mAP0.5 (%)	Params (M)
Fast YOLO^18^	52.7	–
Faster R-CNN-VGG-16^18^	73.2	–
SSD300^18^	79.6	36.1
SSD512^18^	81.6	36.1
SSD512^18^	81.6	36.1
ShuffleNetV2-SSDLite^32^	65.4	**2**.**17**
RefineDet512-VGG-16^33^	83.8	–
RFB Net512-VGG^33^	82.8	–
MobileNetV2-YOLOV4^34^	81.5	46.34
EEEA-Net-C2-YOLOV4^34^	81.8	31.15
YOLOV5s^29^	82.8*	7.06
YOLOV6-N^35^	79.3*	6.4
YOLOV7-Tiny^36^	80.9*	6.2
LKC-Net(Ours)	**84.0**	7.28

The results with * in the table mean that this model is tested in the same environment, and the code comes from open source.

Bold indicates the optimal performance.

Table 2 shows that the mAP0.5 of LKC-Net is increased by 1.2% in comparison with the original YOLOV5s. Compared with the models with the large number of parameters, such as MobileNetV2-YOLOV4 and SSD300, the accuracy of LKC-Net is 2.5% higher than that of MobileNetV2-YOLOV4 and 2.4% higher than that of SSD300. In comparison with the models with similar parameters, such as YOLOV6-N and YOLOV7-Tiny, the detection accuracy of the LKC-Net is 4.7% higher than that of YOLOV6-N and 3.1% higher than that of YOLOV7-Tiny. In summary, LKC-Net achieves the best detection accuracy while maintaining a small number of parameters. Therefore, LKC-Net achieves optimal detection performance.

#### Experiment on MS COCO datasset

Furthermore, LKC-Net is compared with MNetV1-SSDLite^37^, MNetV2-SSDLite^37^, RefineDet512-VGG-16^33^, RFBNet512-VGG^33^, MnasNet-A1-SSDLite^38^, Retina Net640-ResNet-50^39^, YOLOV3-ASFF320^40^, PPYOLO-Tiny416^41^, YOLOV4-Tiny320^42^, YOLOX-Tiny^43^, YOLOV5s^29^,DAMO-YOLO-Ns^44^,DAMO-YOLO-NM^44^, PP-Picodet-M^45^, PP-PicoDet-MV3-large-1x^45^, PP-PicoDet-LCNet-1.5x^45^ EffificientDet-D0^46^(512) and YOLOV7-Tiny640^36^. The accuracy of these algorithms on the MS COCO dataset is shown in Table 3.

**Table 3:** Comparison with different models on MS COCO dataset.

Models	AP@.5:0.95 (%)	Params (M)
MNetV1-SSDLite^37^	22.2	5.10
MNetV2-SSDLite^37^	22.1	**1.30**
RefineDet512-VGG-16^33^	33.0	–
RFBNet512-VGG^33^	33.8	–
MnasNet-A1-SSDLite^38^	23	4.90
RetinaNet640-ResNet-50^39^	37.0	–
YOLOV3-ASFF320^40^	38.1	–
PPYOLO-Tiny416^41^	22.7	4.20
YOLOV4-Tiny320^42^	28.7	5.89
YOLOX-Tiny^43^	32.8	5.1
YOLOV5s^29^	37.2	–
YOLOV7-Tiny640^36^	37.4	6.2
DAMO-YOLO-Ns^44^	32.3	1.41
DAMO-YOLO-Nm^44^	38.2	2.14
PP-Picodet-M^45^	34.3	2.15
PP-PicoDet-MV3-large-1×^45^	35.6	2.80
PP-PicoDet-LCNet-1.5×^45^	36.3	3.10
EffificientDet-D0 (512)^46^	34.6	3.9
LKC-Net (Ours)	**38.4**	7.2

Bold indicates the optimal performance.

Table 3 shows that the mAP0.5:0.95 of LKC-Net model increased by 1.2% compared with the YOLOV5s model. In comparison with the other lightweight YOLO model, it can be seen that the accuracy of LKC-Net is improved by 16.2% compared with MNetV1-SSDLite, 16.3% compared with MNetV2-SSDLite, and 15.4% compared with MnasNet-A1+SSDLite. It is 9.7% more accurate than YOLOV4-Tiny320 and 5.6% more accurate than YOLOX-Tiny. In comparison with other one-stage detection models, LKC-Net is 5.4% better than RefineDet512-VGG-16, 2.5% better than YOLOV6-N, 5.1% better than YOLOV7-Tiny, and 0.3% better than YOLOV3-ASFF320. In Comparsion with the selected SOTA methods, LKC-Net is 6.1% better than DAMO-YOLO-Ns,0.2% better than DAMO-YOLO-Nm, 4.1% better than PP-Picodet-M, 2.8% better than PP-PicoDet-MV3-large-1×, 3.8% better thanEffificientDet-D0. In summary, LKC-Net achieves the best detection accuracy while maintaining a small number of parameters. Therefore, LKC-Net has achieved the best detection performance.

### Validity analysis and ablation experiment

#### Parameters and computation of different convolutions

To verify the improvement of feature capture enhancement block, the changes in the number of parameters and computation cost before and after adding the feature capture enhancement block are computed, as shown in Table 4.

**Table 4:** Comparison of parameters between FCE and standard convolution.

Knernel size	Parameters (M)	GFLOPs (G)	FCE parameters (M)	FCE GFLOPs (G)
1*1+1*1	7.23	16.6	7.23	16.60
17*17+1*1	29.25	34.2	7.19	16.61
17*17+5*5	30.82	39.3	7.21	16.61

Table 4 shows that when the size of the first convolutional block in the neck is increased to 17*17, the number of standard convolutional parameters increases from 7.23M to 29.25M, the number of parameters increases nearly four times, and the calculation amount increases from 16.6M to 34.2M, nearly two times. On this basis, when the size of the second convolution kernel block of the neck is increased to 5*5, the number of parameters is further increased from 29.25M to 30.82M, and the amount of computation is increased from 34.2 to 39.3%. However, when the feature capture enhancement block is replaced, it can be seen that the total parameters and computation cost of the network do not change significantly after increasing the size of the convolution kernel.

#### Different kernel sizes and attention mechanisms

The convolution kernel size of the standard convolution block in the original YOLOV5s is increased for the experiment. The original convolution kernel size is changed to 5*5. Then, the kernel size is gradually increased to obtain the best performance. It is found that the best convolution kernel size is the combination 17*17 of 5*5. The results are shown in Table 5.

**Table 5:** Comparison of different kernel sizes.

Kernel size	mAP0.5 (%)	Kernel size	mAP0.5 (%)
5*5	82.9	19*19	83.6
13*13	83.4	17*17+5*5	83.7
17*17	83.7	17*17+7*7	83.3

Table 5 shows that when the kernel size is increased to 5*5, the detection effect is improved from 82.8 to 83.4% in comparison with the original YOLOV5s model. With the increase of the kernel size, the detection effect is also significantly enhanced, and the detection effect reaches the best (83.7%) when the kernel size is increased to 17*17. When the kernel size is increased to 19*19, the detection effect begins to deteriorate, and reduces the detection effect. The size of the second convolution kernel block also is increased to 5*5, and the model’s accuracy does not increase significantly at the beginning, remaining at 83.7%. When it increases again, the accuracy begins to plummet. Therefore, the optimal convolution kernel size is the combination 17*17 of 5*5.

To compare the influence of standard convolution and depthwise convolution, the comparison experiments for the method with standard convolution and depthwise convolution are conducted, as shown in Table 6.

**Table 6:** Comparison between Depthwise Conv and Standard Conv.

Conv block	Kernel size	Parameters (M)	GFLOPs	mAP0.5 (%)
Standard Conv	17*17+5*5	30.82	39.3	83.8
Depthwise Conv	17*17+5*5	7.2	16.61	83.7

Table 6 shows that when large kernel convolution is used in depthwise convolution, the number of parameters is almost a quarter of standard convolution, the number of parameters is a third of standard convolution, and the final effects of different convolutions are almost the same.

Furthermore, to verify the effect of different attention mechanisms on the model, a series of experiments with different attention mechanisms on the model is constructed, as shown in Table 7.

**Table 7:** Comparison of different attention mechanisms.

Attention	mAP0.5 (%)
FCE	83.7
FCE + CBAM	83.2
FCE + SE	83.2
FCE + CA	83.6
FCE + VRF	83.8

Table 7 shows that different attention mechanisms have different effects on the proposed model, among which the large receptive field attention mechanism has a better effect on detecting the convolution model with a large kernel. The other attention mechanisms, such as CBAM and SE, hinder the improvement of model accuracy. In the network with the addition of the FCE, the network detection accuracy with the addition of CBAM decreases from 83.7% to 83.2%. The accuracy of network detection with the addition of the SE attention mechanism also decreases from 83.7 to 83.2%, and the accuracy of network detection with the addition of the CA attention mechanism decreases from 83.7 to 83.6%. However, when the VRF attention mechanism is used, the accuracy of the model is improved from 83.7 to 83.8%.

Finally, we evaluate the influence of several different loss functions, including GIOU, DIOU and SIOU. The results are shown in Table 8. Table 8 shows that DIOU has a poor fit to the model, resulting in a loss of 0.2% in the proposed model, while GIOU and SIOU both have certain improvement effects on the model, increasing by 0.1% and 0.2% respectively. Therefore, SIOU is selected as the loss function for the model.

**Table 8:** Comparison of different loss function.

Loss function	mAP0.5 (%)
FCE + VRF	83.8
FCE + VRF + GIOU	83.9
FCE + VRF + DIOU	83.6
FCE + VRF + SIOU	84.0

#### Ablation study

To demonstrate the respective roles of different components in the proposed model, including using large kernel convolution, the feature capture enhancement (FCE) block, the vast receptive field (VRF) attention mechanism, and the loss function, the ablation study on the Pascal VOC dataset is carried out. For a finer analysis, the three components are added successively in the ablation experiment, and the improvement on the model is shown in Table 9.

**Table 9:** Results of ablation experiment.

	Large kernel Conv	FCE	VRF	SIoU	mAP0.5 (%)
YOLOV5s					82.8
YOLOV5s	$\checkmark$				83.8
YOLOV5s	$\checkmark$	$\checkmark$			83.7
YOLOV5s	$\checkmark$	$\checkmark$	$\checkmark$		83.8
YOLOV5s	$\checkmark$	$\checkmark$	$\checkmark$	$\checkmark$	**84.0**

Bold indicates the optimal performance.

Table 9 shows that the enlargement of kernel size from 1*1 to 17*17 can improve the network detection performance. Although the accuracy of the model is improved by 1%, the number of parameters and calculation amount are significantly increased, as shown in Table 6. Then, FCE block is used to reduce the number of parameters and calculation amount. On this basis, VRF can enhance the attention of the network model to the channel direction of the feature map, which further improves the accuracy of the network model from 83.7 to 83.8%. Finally, the loss function introduced into the model further improves the detection accuracy of the proposed network from 83.8 to 84.0%.

### Visualization comparison

#### Visualization comparison of receptive field

**Figure 7:**
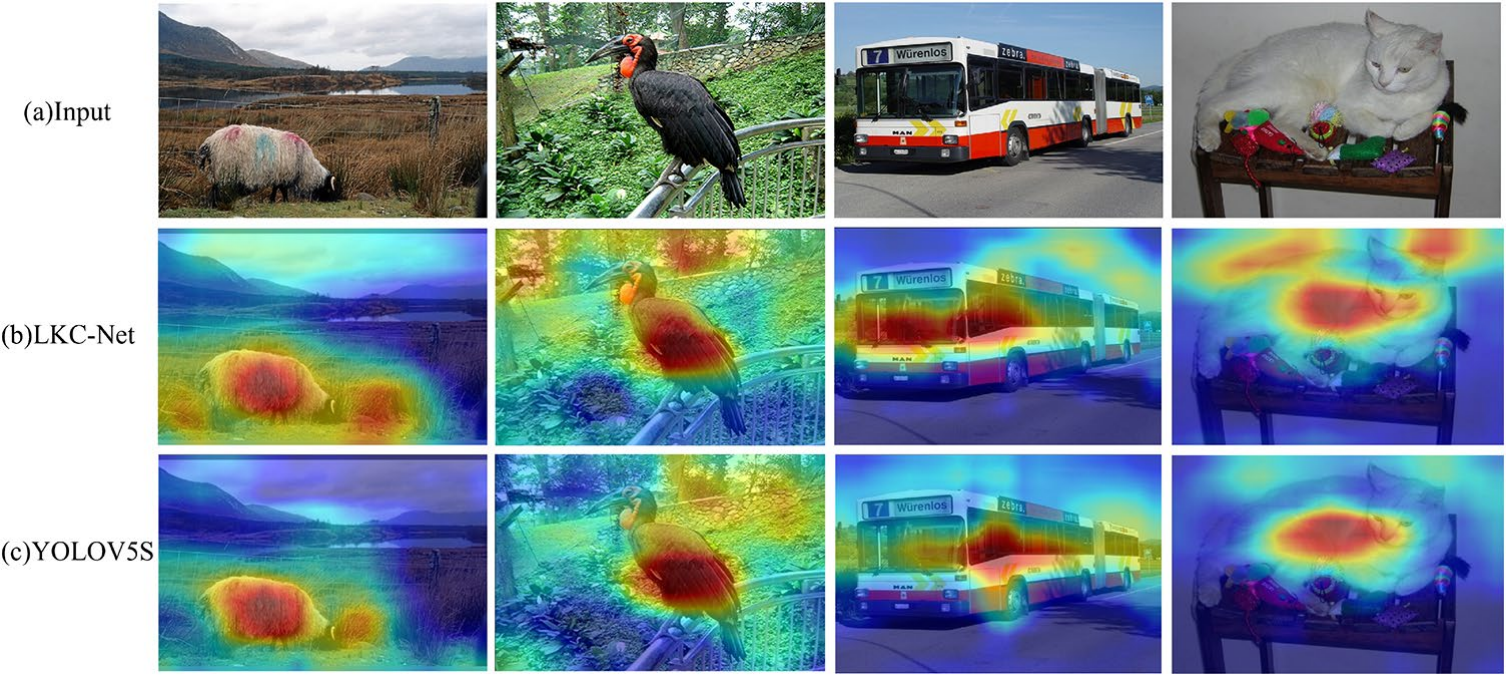
Comparison of receptive field.

The Grad-CAM^47^ visualization method is used to conduct receptive field visualization experiments on the YOLOV5 network and LKC-Net network, as shown in Fig. 7.

Figure 7 shows that the larger receptive field of LKC-Net is reflected in the third line of the heat map compared to the YOLOV5s, which means that the network pays more attention to the input image from a larger vision. As can be seen from Fig. 7, LKC-Net benefits from the large receptive field brought by the large convolution kernel, which makes LKC-Net not only interested in the target itself to be detected but also able to notice the contextual semantic information of the detected object.

#### Visualization experiments of improvement effect

**Figure 8:**
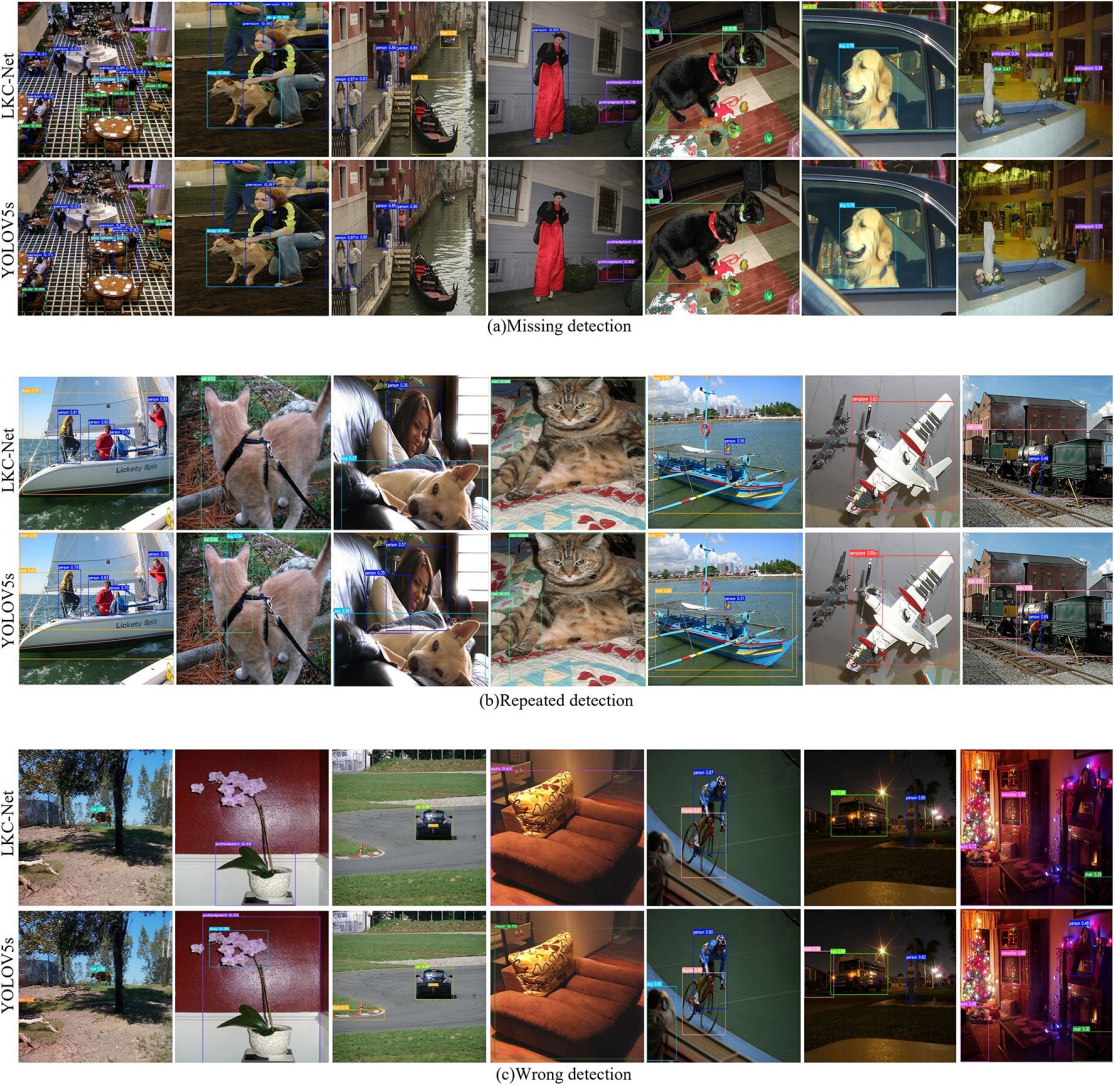
Performance improvements of LKC-Net.

To verify the improvement on the original YOLOV5s network, the visualization experiments of the improvement effect are performed, and the improvements of LKC-Net are shown in Fig. 8.

Figure 8a show that the LKC-Net network can solve the problem of missing detection compared with the YOLOV5s network. The first group of pictures shows the missed chairs, the second group of pictures shows the missed dogs, the third group of pictures shows the missing boats, the fourth group of pictures shows the missing people, the fifth group of pictures shows the missed cats, the sixth group of pictures shows the missed cars, and the seventh group of pictures shows the missed chairs and potted plants. Figure 8b show that the LKC-Net network can solve the problem of YOLOV5s repeated detecting large objects in the image.The first and fifth sets of pictures show the repeated detection of ships, the second and fourth sets of pictures show the repeated detection of cat, the third set of pictures show the repeated detection of people, the sixth set of pictures show the repeated detection of planes, and the seventh set of pictures show the repeated detection of trains. Figure 8c show that LKC-Net can improve the YOLOV5s’s wrong detection problem. The first set of images corrected that the model detected tree trunk as bird, the second set of images corrected that the model detected potted plant as dog, the third set of images corrected that the model detected runway track as boat, the fourth set of images corrected that the model detected sofa as chair, the fifth set of images corrected that the model detected person as dog, the sixth set of images corrected that the model detected house as train, and the seventh set of images corrected that the model detected clock as person. Therefore, LKC-Net can effectively improve the problems of missing detection, wrong detection, and repeated detection by virtue of the large receptive field brought by the large kernel convolution.

#### Visualization of limitation

**Figure 9:**
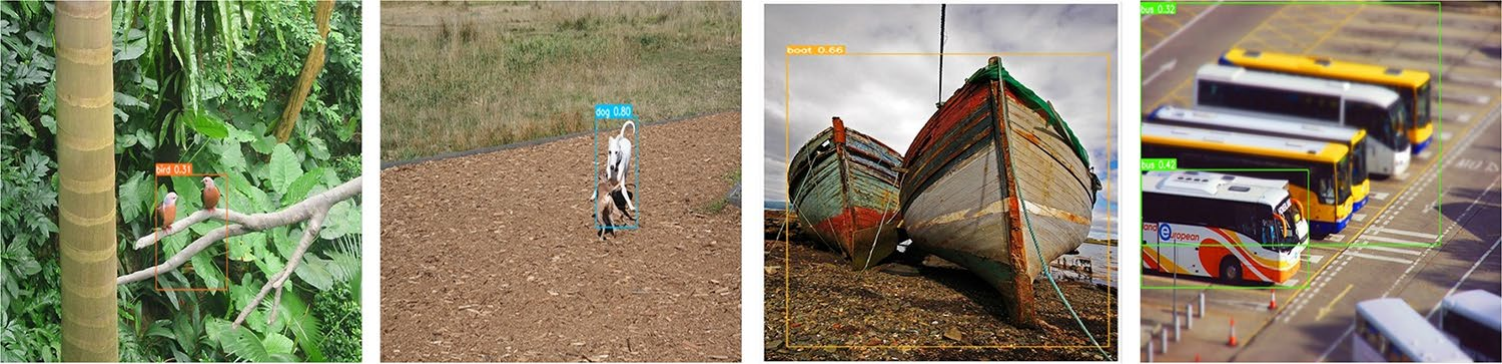
Limitation of LKC-Net.

Although LKC-Net has significant performance improvements compared with the baseline model in different respects, there is still the limitation of the proposed method. In certain detection scenarios, when the distance between objects of the same class is small, it can cause the model to mistakenly recognize two objects as one object, as shown in Fig. 9. In the first image, the model identified two birds as one bird. The same problem occurs in the other three images.

## Conclusion

In this paper, an object detection network based on a large kernel convolutional neck network is proposed. Firstly, the feature capture enhancement block based on large kernel convolution is proposed to improve the semantic feature capturing ability, and the depth convolution is used to reduce the number of parameters. Then, the vast receptive field attention mechanism is constructed to enhance channel direction information extraction ability. The experimental results demonstrate the constructed attention mechanism is more compatible with the proposed backbone than other existing attention mechanisms. Third, the loss function is improved by introducing the SIoU to overcome the angle mismatch problem between the ground truth and prediction box. Pascal VOC and MS COCO datasets are used to compare the object detection performance of LKC-Net with other existing models. The quantitative evaluation results demonstrate that LKC-Net can achieve the best object detection performance in terms of accuracy while maintaining a small number of parameters. The qualitative evaluation results demonstrate that LKC-Net benefits from the large kernel convolution structure, which enhances contextual semantic information extraction ability and overcomes the wrong detection, missing detection, and repeated detection problems. In future work, we will focus on making the proposed network more lightweight and adjusting the convolution kernel size to further enhance object detection performance.In future work, we will focus on some promising directions worth pursuing including: make the proposed network more lightweight; adjuste the convolution kernel size to further enhance performance; combine the proposed method with different baselines.

## Data Availability

The datasets used in this study are publicly available. Pascal VOC dataset is available on the official website: http://host.robots.ox.ac.uk/pascal/VOC/voc2012/. MS COCO dataset is available on the official website: https://cocodataset.org/#download. The images in this manuscript are all from publicly available Pascal VOC and MS COCO datasets.
